# Early ERCP in Biliary Pancreatitis?

**DOI:** 10.1155/1998/18692

**Published:** 1998-12

**Authors:** Margaret D. Finch, John P. Neoptolemos

**Affiliations:** Department of Surgery University of Liverpool 5th Floor UCD Building, Daulby Street Liverpool L69 3GA UK


*HPB Surgery*, 1998, Vol. 11, pp. 129-139
Reprints available directly from the publisher
Photocopying permitted by license only

© 1998 OPA (Overseas Publishers Association) N.V.
Published by license under
the Harwood Academic Publishers imprint,
part of The Gordon and Breach Publishing Group.
Printed in Malaysia.

### HPB INTERNATIONAL

**EDITORIAL & ABSTRACTING SERVICE**

**JOHN TERBLANCHE EDITOR**

Department of Surgery,
Medical School · Observatory 7925
Cape Town · South Africa
Telephone: 27-21-406-6204
Telefax 27-21-448-6461
E-mail: jterblan@uctgsh1.uct.ac.za

### Early ERCP in Biliary Pancreatitis?

### ABSTRACT

*Folsch, U. R., Nitsche, R., Ludtke, R., Hilgers, R. A. and Creutzeldt, W., The Germany Study Group on Acute Biliary Pancreatitis. (1997) Early ERCP and papillotomy compared with conservative treatment for acute biliary pancreats. New England Journal of Surgery;* **336**, *237-242.*

***Background:*** **The role of early endoscopic retrograde cholangiopancreatography (ERCP) and papillotomy in the treatment of patients who have acute biliary pancreatitis without obstructive jaundice is uncertain.**
***Methods:*** **We conducted a prospective, multicenter study in which 126 patients were randomly assigned to early ERCP (within 72 hours after the onset of symptoms) and endoscopic papillotomy for the removal of stones in the common bile duct, when appropriate, and 112 patients were assigned to conservative treatment. In the conservative-treatment group, ERCP was performed within three weeks if signs of biliary obstruction or sepsis developed. Overall mortality, mortality due to pancreatitis, and complications were compared in the two groups.**
***Results:*** **Early ERCP was successful in 121 of the 126 patients in the invasive-treatment group. Endoscopic papillotomy was performed to remove bile-duct stones in 58 patients; stones were successfully extracted in 57. ERCP was performed in 22 of the 112 patients in the conservative-treatment group; papillotomy for stone removal was successful in 13 patients. Fourteen patients in the invasive-treatment group and 7 in the conservative-treatment group died within three months (P = 0.10); 10 patients in the invasive-treatment group and 4 in the conservative-treatment group died from acute biliary pancreatitis (P = 0.16). The overall rate of complications was similar in the two groups, but patients in the invasive-treatment group had more severe complications. Respiratory failure was more frequent in the invasive-treatment group, and jaundice was more frequent in the conservative-treatment group.**

***Conclusions*:** **In patients with acute biliary pancreatitis but without obstructive jaundice, early ERCP and papillotomy were not beneficial. (*N. Engl. J. Med.*, 1997; 336; 237-42.)**

*Keywords:* Biliary pancreatitis, pancreatitis, ERCP, sphincterotomy

### PAPER DISCUSSION

The report by Fölsch and colleagues represents the fourth published randomised trial of early ERCP and endoscopic sphincterotomy (ES) versus conservative management for acute biliary pancreatitis [1-3]. The premise of the study design is that patients with coexisting biliary obstruction have already been shown to benefit from early endoscopic sphincterotomy. The study therefore aimed to ascertain benefit for early sphincterotomy in acute pancreatitis in the absence of biliary obstruction and concluded that it was of no benefit. Several flaws in the study, however, make such a conclusion insupportable.

Antibiotics were not routinely used, although their use was a feature of at least two of the previous trials [1, 2], an important difference given recent evidence for the positive influence of antibiotics on outcome in acute pancreatitis [4, 5].

Patients were not prospectively stratified for predicted severity. This makes any subgroup analysis statistically meaningless because it is retrospective. The modified Glasgow severity criteria were used which can only be determined 48 hours following admission, yet the median time for intervention was 36 hours. Therefore if ES was performed sooner than 48 hours a severe case could have been converted to the mild category.

Unlike the other randomised trials which were single centre studies [1,2,3], this study involved 22 centres meaning that there were only 2 patients entered per centre per year (range of total entered 6–29 per centre). This is difficult to understand as patients were apparently entered consecutively.

No explanation is given as to why 17 patients in the treatment group developed acute cholangitis (compared to 13 cases in the control group). Since no other study of ES in acute pancreatitis approaches such figures we can only presume that inadequate ES was common.

The high mortality in the treatment group also runs contrary to any previously published experience of ES in acute pancreatitis. There were 12 hospital deaths but only 26 severe cases, giving a mortality for this group of 46%, or 32% if all 16 patients with undefined severity are included. Since deaths from ES in mild acute pancreatitis are rare, the high mortality raises serious questions regarding the standard of ES performed and the overall management of acute pancreatitis.

In order to detect a mortality difference of 2% to 8% the authors calculated that 380 patients were required (190 in each group) for $\alpha$=0.05 and a power of 80%. The trial however was stopped after only 238 patients were entered (63% of the target), so the survival objectives were not reached. Therefore the study was seriously underpowered and cannot support the authors' own conclusions.

The authors point out that the study from Hong Kong [3] included some patients with nonbiliary causes of acute pancreatitis. It is true that only 65% of the patients had confirmed gallstones and nearly one third of the conservative group had ERCP/ES and mostly within 72 hours. Parallel analysis of the Hong Kong and Leicester studies, however, including only patients with confirmed gallstones shows remarkably similar results including a reduction in local and systemic complications and death following intervention (Tab. I).

In the *Discussion* mention was made of a randomised study of surgery [6] which reached the same conclusions as those of the authors. Indeed that study suffered from similar flaws to this one including (i) post hoc severity stratification and therapeutic intervention before predicted severity could be determined and (ii) the

TABLE I Results of the Leicester [1] and Hong Kong [2] studies of ERCP and ES in acute pancreatitis comparing only those with proven gallstones comparing mild with severe cases

| City Treatment | Numbers of Patients: Mild | Complications: Local | Complications: Systemic | Deaths | Severe | Complications: Local | Complications: Systemic | Deaths |
|---|---|---|---|---|---|---|---|---|
| **HONG KONG** | | | | | | | | |
| ERCP/ES | 34 | 4 (12%) | 2 (6%) | 0 | 30 | 3 (10%)[b] | 3 (10%)[f] | 1 (3%)[j] |
| Conventional | 35 | 1 (3%) | 5 (14%)[4][a] | 0 | 28 | 8 (29%)[c] | 16 (57%)[g] [8][a] | 5 (18%)[k] |
| **LEICESTER** | | | | | | | | |
| ERCP/ES | 28 | 3 (11%) | 1(4%) | 0 | 22 | 3 (14%)[d] | 1 (5%)[h] | 0[l] |
| Conventional | 29 | 4 (14%) | 0 | 0 | 24 | 6 (25%)[e] | 9 (38%)[i] | 3(13%)[m] |

[a] numbers of patients with biliary sepsis. Statistical differences for the mild groups of patients were not significant.
(b+d) vs (c+e): $p < 0.05$, (f+h) vs (g+i): $p < 0.01$, (j+l) vs (k+m): $p < 0.05$.

use of a surgical technique which could not adequately decompress the common channel (supraduodenal exploration with insertion of a T-tube).

The weight of evidence from three previous trials support the use of emergency ES in patients with severe acute pancreatitis due to gallstones irrespective of concomitant acute cholangitis or obstructive jaundice alone. This requires ES to be performed by properly trained personnel [7] and can only be of value if there is a high standard of overall management [1–6].

### References

[1] Neoptolemos J. P., London, N. J., James, D., Carr-Locke, D. L., Bailey, I. A. and Fossard, D. P. (1988). Controlled trial of urgent endoscopic retrograde cholangiopancreatography and endoscopic sphincterotomy versus conservative treatment for acute pancreatitis due to gallstones. *Lancet*, **2**, 979–83.

[2] Nowak, A., Nowakowska-Dulaw, E., Marek, T. A. and Rybicka, J. (1995). Final results of the prospective, randomised, controlled study on endoscopic sphincterotomy versus conventional treatment in acute biliary pancreatitis. *Gastroenterology*, **108**, A380 (Abstract).

[3] Fan, S. T., Lai, C. S., Mok, F. P. T., Lo, C. M., Rheng, S. S. and Wong, J. (1993). Early treatment of acute biliary pancreatitis by endoscopic papaillotomy. *New England Journal of Medicine*, **328**, 228–32.

[4] Luiten, E. J. T., Hop, W. L. J., Lange, J. F. and Browning, H. A. (1995). Controlled clinical trial of selective decontamination for treatment of severe acute pancreatitis. *Ann. Surg.*, **222**, 57–65.

[5] Sainio, V., Kempainen, E., Paolakkainen, P., Taavitsainen, M., Kivisaari, L., Valtonen, V., Haapiainen, R., Schrder, T. and Kivilaakso, E. (1995). Early antibiotic treatment in acute necrotising pancreatitis. *Lancet*, **346**, 663–67.

[6] Kelly, T. R. and Wagner, D. S. (1988). Gallstone pancreatitis: a prospective randomised trial of the timing of surgery. *Surgery*, **104**, 600–5.

[7] Freeman, M. L., Nelson, D. B., Sherman, S. *et al.* (1996). Complications of endoscopic biliary sphicterotomy. *N. Engl. J. Med. C.*, **335**, 909–18.

Margaret D Finch and
Professor John P Neoptolemos
Department of Surgery
University of Liverpool
5th Floor UCD Building
Daulby Street
Liverpool L69 3GA
United Kingdom

### Sclerotherapy or Banding for Oesophageal Varices?

### ABSTRACT

*Sarin, S. K., Govil, A., Jain, A. K., Guptan, R. C., Issar, S. K., Jain, M. and Murthy, N. S. (1997) Prospective randomised trial of endoscopic sclerotherapy versus variceal band ligation for esophageal varices: influence on gastropathy, gastric varices and variceal recurrence. Journal of Hepatology, 26, 826–832.*

*Background/Aims:* **Endoscopic variceal ligation and endoscopic sclerotherapy are both recommended for the prevention of variceal rebleeding. To compare their prevention of variceal rebleeding. To compare their efficacy, their influence on gastric varices and the development of portal gastropathy, 95 patients with variceal bleeding were studied.**

*Methods:* **The patients were randomized to receive weekly endoscopic sclerotherapy using alcohol ($n = 48$) or endoscopic variceal ligation ($n = 47$). The endoscopic sclerotherapy and endoscopic variceal ligation groups were comparable in etiology, severity of liver disease and grade of varices.**

*Results:* **In the arrest of acute bleed, endoscopic sclerotherapy and endoscopic variceal ligation were comparable (86% vs. 80%, $p =$ ns). Endoscopic variceal ligation as compared to endoscopic sclerotherapy, obliterated esophageal varices in fewer sessions (4.1±1.2 vs. 5.2±1.8, $p < 0.01$) and a shorter time (4.4±1.3 vs. 6.9±3.4 wk, $p < 0.01$). Three (6.4%) patients bled after endoscopic variceal ligation and 10 (20.8%) after endoscopic sclerotherapy ($p<0.05$). The actuarial percentage of variceal recurrence during a follow-up of 8.5±4.4 months, was higher after endoscopic variceal ligation than endoscopic sclerotherapy (28.7% vs 7.5%, $p < 0.05$). Esophageal stricture formation after endoscopic sclerotherapy occurred in five (10.4%) patients, but in none after endoscopic variceal ligation. Significantly more patients developed gastropathy after endoscopic sclerotherapy than ligation (20.5% vs. 2.3%; $p = 0.02$). Endoscopic sclerotherapy (52%) and endoscopic variceal ligation (59%) were equally effective in obliterating the lesser curve gastric varices. Six patients died: three in each group.**

*Conclusion:* **(i) Endoscopic sclerotherapy and endoscopic variceal ligation were equally effective in controlling acute bleed; (ii) endoscopic ligation achieved variceal obliteration faster and in fewer treatment sessions; (iii) endoscopic variceal ligation had a significantly lower rate of develop-**

